# Supplementary material for: Multidisciplinary team healthcare professionals’ perceptions of current and optimal acute rehabilitation, a hip fracture example A UK qualitative interview study informed by the Theoretical Domains Framework
Source: PLoS One. 2022 Nov 18;17(11):e0277986. doi: 10.1371/journal.pone.0277986 (PMC9674178; doi:10.1371/journal.pone.0277986)
Supplement: S1 Table — (DOCX) [file pone.0277986.s001.docx]

**Supporting Information Table 1. Questions and prompts mapped to the Theoretical Domains Framework domains and constructs**

| **TDF Domains and definition** | **TDF constructs** | **Questions and prompts [Constructs]** |
| --- | --- | --- |
| Knowledge  (An awareness of the existence of something) | Knowledge of condition/scientific rationale  Procedural knowledge  Knowledge of task environment  Knowledge of scientific rationale | **Q1** Please could you tell me about your role in rehabilitation after hip fracture?  **Q2** What types of patients do you see with hip fracture?  Prompt: how do you work with these/different patients?  **Q3a** What is the current model for rehabilitation for patients after hip fracture in your hospital? [Procedural knowledge, knowledge of task environment, knowledge of scientific rationale]  Prompt: what is the rationale for the current model?  Prompt: How feasible is it for you to implement this model? / What are the facilitators and barriers to successful delivery of the model? e.g. Patient facilitators/barriers? e.g., Resource facilitators/barriers? / How do you manage working within the model?  Q3b What are your thoughts on this model?  Prompt: How confident are you in your hospitals model? / How confident are you that you can deliver the model effectively?  *Positive / Facilitators*  Prompt: What in particular makes the model work so well?  Prompts: What helps you and your colleagues to successfully deliver the model?  Prompt: What advice would you give to another hospital hoping to make changes?  *Negative / Barriers*  Prompt: What challenges do you face when implementing this model? / Can you provide an example of a situation where it was challenging to deliver the model effectively? Does this occur often?  Prompt: How would you change the model? / What have you done to change the model? How do you overcome this challenge? How have you addressed this issue?  Prompt: Are you aware of models in other hospitals that you would like to implement at your hospital?  *Other Staff*  Prompt: How do you think your thoughts compare to other healthcare professionals at your hospital? / To what extent do you think your colleagues share your views?  Prompt: To what extent do you believe your model is interdisciplinary? Can you provide some examples?  Prompt: To what extent do you think the responsibility for successful rehabilitation falls on some professionals more than others? Can you provide some detail why?  Prompt: How do you think your thoughts compare to other healthcare professionals at your hospital? / To what extent do you think your colleagues share your views?  *Monitoring Model Outcomes*  Prompt: How do you keep up to date with changes in aspects of care delivery for patients after hip fracture?  Prompt: How do you monitor success?  Prompt: What changes could you implement?  **Q4** To what extent is the model provided for all patients consistently? OR How does care vary for different types of patients / on patients depending on how they present OR How do you accommodate for different patient need?  Prompt: How does your model accommodate for patients with hip fracture and cognitive impairment? / How confident are you your hospitals model accommodates / can be delivered effectively for those with cognitive impairment?  Prompt: How feasible is it for you to implement this model with patients? / What challenges do you face when implementing this model for all patients?  *Training*  Prompt: What training is available at your site for healthcare professionals working with patients with hip fracture? Is this training formal or informal?  What is involved in ‘X’ training?  *Change*  Prompt: How would you change the model? / What could be put in place to change the model if needed?  **Q5** What do you think the role of other healthcare professionals is in rehabilitation after hip fracture? [Procedural knowledge]  Prompt: How do you see your role fitting in within the team? / How does your role differ from other healthcare professionals in the team? / How do you think other healthcare professionals would describe your role?  Prompt: What is involved in your role + what are the facilitators and barriers to this?  Prompt: What aspects of acute rehabilitation do you identify as being part of your role as a …?  Prompt: How do you interact with other healthcare professionals in the management of patients with hip fracture? |
| Skills  (An ability or proficiency acquired through practice) | Skills  Skills development  Competence  Ability  Interpersonal skills  Practice  Skill assessment | **Q2** Can you describe the types of patients that you see with hip fracture?  Prompt: how do you work with these/different patients?  **Q3b** What are your thoughts on this model? [Ability, Practice]  Prompt: How do you manage working within the model?  **Q4** To what extent is the model provided for all patients consistently? [Competence, ability, practice]  Prompt: What training is available at your site for healthcare professionals working with patients with hip fracture?  **Q5** What do you think the role of other healthcare professionals is in rehabilitation after hip fracture? [Interpersonal skills]  Prompt: How do you interact with other healthcare professionals in the management of patients with hip fracture?  **Q6** What do you think the role of patients and their carers is in rehabilitation after hip fracture? [Professional boundaries]  Prompt: How do you interact with carers in the management of patients with hip fracture?  Prompt: What do you consider a reasonable contribution to expect from a caregiver? |
| Social/ Professional role  and identity  (A coherent set of behaviours and displayed personal qualities of an individual in a social or work setting) | Professional identity  Professional role  Social identity  Identity  Professional boundaries  Professional confidence  Group identity  Leadership  Organizational commitment | **Q1** Please could you tell me about your role in rehabilitation after hip fracture? [Professional role, identity]  **Q4** To what extent is the model provided for all patients consistently? [Professional confidence]  Prompt: How does your role accommodate for patients with hip fracture and cognitive impairment?  **Q5** What do you think the role of other healthcare professionals is in rehabilitation after hip fracture? [Professional boundaries, group identity, organisational commitment]  Prompt: How do you see your role fitting in within the team?  Prompt: How does your role differ from other healthcare professionals in the team?  Prompt: What are your thoughts on professional boundaries for multidisciplinary team members? (crossover of scope of practice)  **Q6** What do you think the role of patients and their carers is in rehabilitation after hip fracture? [Professional boundaries]  Prompt: How do you interact with carers in the management of patients with hip fracture?  Prompt: What do you consider a reasonable contribution to expect from a caregiver? |
| Beliefs about capabilities  (Acceptance of the truth, reality or validity about an ability, talent or facility that a person can put to constructive use) | Self-confidence  Perceived competence  Self-efficacy  Perceived behavioural control  Beliefs  Self-esteem  Empowerment  Professional confidence | **Q1** Please could you tell me about your role in rehabilitation after hip fracture? [Beliefs]  **Q3b** What are your thoughts on this model? [Perceived behavioural control, Professional Confidence]  Prompts: What helps you and your colleagues to successfully deliver the model?  Prompt: How confident are you in your hospitals model?  Prompt: How confident are you that you can deliver the model effectively?  Prompt: Can you provide an example of a situation where it was challenging to deliver the model effectively? Does this occur often?  **Q4** To what extent is the model provided for all patients consistently? [Perceived behavioural control, professional confidence]  Prompt: How confident are you your hospitals model accommodates for those with cognitive impairment?  Prompt: How confident are you that you can deliver the model effectively for patients with cognitive impairment? |
| Optimism  (The confidence that things will happen for the best or that desired goals will be attained) | Optimism  Pessimism  Unrealistic optimism  Identity | **Q3b** What are your thoughts on this model? [optimism, pessimism, unrealistic optimism]  Prompt: How do you think your thoughts compare to other healthcare professionals at your hospital?  **Q4** To what extent is the model provided for all patients consistently? [optimism, pessimism, unrealistic optimism]  Prompt: How do you think your thoughts compare to other healthcare professionals at your hospital? |
| Beliefs about consequences  (Acceptance of the truth, reality, or validity about outcomes of a behaviour in a given situation) | What are the benefits/negative aspects of the behaviour (model)?  Beliefs  Outcome expectancies Characteristics of outcome expectancies  Anticipated regret  Consequences | **Q3b** What are your thoughts on this model? [Beliefs, outcome expectancies, characteristics of outcome expectancies, anticipated regret, consequences]  Prompt: What in particular makes the model work so well?  Prompt: What advice would you give to another hospital hoping to make changes?  Prompt: What has been done to change the model?  Prompt: How would you change the model?  Prompt: What have you done to change the model?  Prompt: Are you aware of models in other hospitals that you would like to implement at your hospital?  **Q4** To what extent is the model provided for all patients consistently? [Beliefs, outcome expectancies, characteristics of outcome expectancies, anticipated regret, consequences]  Prompt: How would you change the model? |
| Reinforcement (Increasing the probability of a response by arranging a dependent relationship, or contingency, between the response and a given stimulus) | Rewards (proximal/distal, valued/not valued, probable/improbable) Incentives  Punishment  Consequents  Reinforcement  Contingencies  Sanctions | **Q4** To what extent is the model provided for all patients consistently? [reinforcement, consequents, contingencies]  Prompt: What could be put in place to change the model if needed? |
| Intentions  (A conscious decision to perform a behaviour or a resolve to act in a certain way | Stability of intentions  Stages of change model Transtheoretical model and stages of change | **Q3b** What are your thoughts on this model? [Stability of intentions]  Prompt: How have your thoughts on the model changed over time?  Prompt: Have you always felt this way about the model?  **Q4** To what extent is the model provided for all patients consistently? [Stability of intentions]  Prompt: Have you always felt this way about the model? |
| Goals  (Mental representations of outcomes or end states that an individual wants to achieve) | Goals (distal/proximal)  Goal priority  Goal/target setting  Goals (autonomous/controlled) Action planning  Implementation intention | **Q3a** What is the current model for rehabilitation for patients after hip fracture in your hospital? [Implementation intention]  Prompt: How feasible is it for you to implement this model?  **Q3b** What are your thoughts on this model? [Goals/target setting, implementation intention]  Prompt: How feasible is it for you to implement this model?  Prompt: What challenges do you face when implementing this model?  **Q4** To what extent is the model provided for all patients consistently? [Implementation intention]  Prompt: How feasible is it for you to implement this model?  **Q7** What do you believe is the goal of the model of rehabilitation at your hospital? [Goals]  Prompt: From the perspective of hospital management? And/or healthcare professionals? How do you feel about these goals? |
| Memory, attention, and decision processes  (The ability to retain information, focus selectively on aspects of the environment and choose between two or more alternatives) | Is X something they usually do? Will they think to do X? Are there reminders in place?  Memory  Attention  Attention Control  Decision making  Cognitive overload/tiredness | **Q3b** What are your thoughts on this model? [memory]  Prompt: How do you keep up to date with changes in aspects of care delivery for patients after hip fracture? |
| Environmental context and resources  (Any circumstance of a person’s situation or environment that discourages or encourages the development of skills and abilities, independence, social competence and adaptive behaviour) | Are there sufficient resources to do the behaviour? If not, what is missing?  Environmental stressors  Resources/material resources  Organizational culture/climate  Salient events/critical incidents  Person x environment interaction  Barriers and facilitators | **Q3b** What are your thoughts on this model? [Environmental stressors, resources/material resources, organisational culture/climate, person x environment interaction, barriers and facilitators]  Prompt: How feasible is it for you to implement this model?  Prompt: What challenges do you face when implementing this model?  Prompt: What are the facilitators and barriers to successful delivery of the model?  **Q4** To what extent is the model provided for all patients consistently? [Environmental stressors, resources/material resources, organisational culture/climate, person x environment interaction, barriers and facilitators]  Prompt: How feasible is it for you to implement this model?  Prompt: What challenges do you face when implementing this model for all patients? |
| Social influences  (Those interpersonal processes that can cause individuals to change their thoughts, feelings, or behaviours) | Who influences the decision to perform the behaviour?  Social pressure  Social norms  Group conformity  Social comparisons  Group norms  Social support  Power  Intergroup conflict  Alienation  Group identity  Modelling | **Q3b** What are your thoughts on this model? [Social norms, group conformity, social comparisons, group norms, social support, power, intergroup conflict, alienation, group identity]  Prompt: To what extent do you think your colleagues share your views?  Prompt: To what extent do you believe you model is interdisciplinary? Can you provide some examples?  **Q4** To what extent is the model provided for all patients consistently? [Social norms, group conformity, social comparisons, group norms, social support]  Prompt: To what extent do you think your colleagues share your views?  **Q5** What do you think the role of other healthcare professionals is in rehabilitation after hip fracture?  [Group conformity, group norms, social support, power, intergroup conflict, alienation, group identity]  Prompt: How do you think other healthcare professionals would describe your role?  **Q6** What do you think the role of patients and their carers is in rehabilitation after hip fracture? [Social support]  Prompt: What do you consider a reasonable contribution to expect from a caregiver? |
| Emotions  (A complex reaction pattern, involving experiential, behavioural, and physiological elements, by which the individual attempts to deal with a personally significant matter or event) | How does emotion affect the behaviour? Is X stressful?  Fear  Anxiety  Affect  Stress  Depression  Positive/negative affect  Burn-out | **Q3b** What are your thoughts on this model? [Stress, burn-out]  Prompt: How feasible is it for you to implement this model for all patients?  Prompt: To what extent do you think the responsibility for successful rehabilitation falls on some professionals more than others? Can you provide some detail why?  **Q8** What are the emotional impacts of delivering your hospital model? [Stress, burn-out, Positive/negative affect]  Prompt: How do you cope with the emotional impact / What support is available to you? |
| Behavioural regulation  (Anything aimed at managing or changing objectively observed or measured actions) | What steps are taken to ensure behaviour is performed?  Self-monitoring  Breaking habit  Action planning | **Q3b** What are your thoughts on this model? [Self-monitoring, action planning]  Prompt: How do you monitor success?  Prompt: What changes could you implement?  **Q4** To what extent is the model provided for all patients consistently? [Self-monitoring, action planning]  Prompt: How do you monitor success?  Prompt: What changes could you implement? |

TDF: Theoretical Domains Framework
