# Supplementary material for: Multidisciplinary team healthcare professionals’ perceptions of current and optimal acute rehabilitation, a hip fracture example A UK qualitative interview study informed by the Theoretical Domains Framework
Source: PLoS One. 2022 Nov 18;17(11):e0277986. doi: 10.1371/journal.pone.0277986 (PMC9674178; doi:10.1371/journal.pone.0277986)
Supplement: S1 Appendix — (DOCX) [file pone.0277986.s002.docx]

# **Supporting Information Appendix I**

Audit trail of phases of qualitative data analysis process with examples.

## **Phase 1: Initial and axial coding from the qualitative data in NVivo (version 12)**

Codes from the data:

Advocacy (for patients, resources)

Barriers to implementation​

Barriers external to the health system​

Communication

With patients and carers

With team, other services in community

Ways of communicating, tools, meetings

Congruence of opinion​

Customization of care to the patient​

Emotional impact to staff​

Family and carers

Education, expectations from rehabilitation​

Feasibility of model for applying to all patients​

Goals of model​

Improvements​

Improvements external to the health system​

Managing comorbidities (cognitive impairment; other health conditions)​

Model outline​

Model rationale​

Monitoring success/ outcomes​ (e.g. audits)

Leadership

Opinion on model​

Strengths of model​

Weaknesses of model​

Other staff's role​

Own role​

Pandemic​

Patients

Patient engagement​

Environment of hospital and resources

Patients for whom model does not work

Education, expectations from rehabilitation

Relations with other staff​

Linkages (outside one’s immediate team; referrals; community care; charities)​

Team culture (work climate)​

Team motivation

Teamwork (ways of working together with other staff)​

Resources in setting​

Resources to help more vulnerable patients

Provision over the weekend

Resources outside the health system (charities, social care etc.)​

Responsibilities​

Systemic problems (coming from the way the health system currently is)​

Training for staff

Role of other professionals

Working with different type of patients (e.g. those living with dementia)

Medical, fracture, medications

## **Phase 2: Categorising and identifying clusters of codes into conceptual themes comparing perspectives across professional groups using the “one sheet of paper method”** approach to synthesise into four themes**

Applying model to practice​

​Barriers to implementation​

Barriers external to the health system​

Opinion on model​

Strengths of model​

Weaknesses of model​

Feasibility of model for applying to all patients​

Goals of model​

Improvements​

Improvements external to the health system​

Model outline​

Model rationale​

Work processes​

Advocacy (for patients, resources)

Customisation of care to the patient​

Monitoring success/ outcomes​ (e.g. audits)

Managing comorbidities (cognitive impairment; other health conditions)​

Team and work environment​

Communication

With team, other services in community

Ways of communicating, tools, meetings

Congruence of opinion​

Emotional impact to staff​

Leadership

Other staff's role​

Own role​

Pandemic​

Relations with other staff​

Linkages (outside one’s immediate team; referrals; community care; charities)​

Team culture (work climate)​

Team motivation

Teamwork (ways of working together with other staff)​

Systemic problems (coming from the way the health system currently is)​

Responsibilities​

Resources in setting​

Resources to help more vulnerable patients

Provision over the weekend

Resources outside the health system (charities, social care etc.)​

Training for staff

Role of other professionals

Working with different type of patients (e.g. those living with dementia)

Medical, fracture, medications

Working with patients and family/carers​

Communication

With patients and carers

Patients

Patient engagement​

Environment of hospital and resources

Patients for whom model does not work

Education, expectations from rehabilitation

Family and carers​

Education, expectations from rehabilitation​

**Phase 3: Mapping themes to TDF domains**

Conceptualising a model of rehabilitative practice

Across services, recurring factors perceived to facilitate optimal rehabilitation (by at least 3 participants) included: teams working well together and supportive consultants and senior management who encouraged improvements to current rehabilitation services (*Social Influences, Social/professional role and identity*), organisational systems for patient notes and to prompt assessments, access to specialised professionals or services (e.g. orthogeriatricians, dieticians, specialised wards), having responsibility over patients’ rehabilitation journey (e.g. deciding on referral pathway or discharge criteria), or providing activities to engage patients in rehabilitation *(Memory, attention and processes, Environmental context and resources, Belief about capabilities, Belief about consequences).*

Where individual participants thought that the model fell far below expectations, this was related to services undergoing significant organisational changes, or shortage and fluctuation of resources such as financial provisions and staffing (*Environmental context and resources*)

*“I think if we’re well staffed we can meet you know, and certainly and do pretty well with the audit and see people quickly, but I think as soon as we’re pressured certainly over the winter months it can be really difficult and if we don’t have the staff often it doesn’t become as high a priority as people that are actually needing to go home that day.” (P17, Band 6 OT)*

Competing professional and organisational goals

Participants commonly commented on a mismatch between the flexibility required to adjust to individual needs (*Skills*) and the organisational goals for a standardised, pre-set model for rehabilitation after hip fracture (*Social/professional role and identity*). This was often reflected by healthcare professional goals of a good foundation for functional recovery on discharge, and organisational goals for discharge home as soon as possible (*Goals*). These competing goals sparked frustrations with participants emphasising the challenges of making a one-size-fits-all model work for the diverse scope of patients that they see with hip fracture (*Intentions*):

*“[I]n the acute service it’s so driven towards just getting someone out of hospital that you can sometimes lose sight of what, what that individual needs as such.” (P16, Band 7 physiotherapist)*

*“[A]ny models are set up for the majority, not for the individual patient, despite everyone aiming to be patient-centred.” (P10, consultant ortho-geriatrician)*

Engaging teams in collaborative practice

For most (n =17), the collaborative nature of their work was underscored in the discussion of their own role and others’ perceived role in rehabilitation (*Professional role and identity*). Participants often commented on perceived unique and overlapping areas of their professional practice and how the engagement of each health professional may vary depending on the needs of an individual patient (*Skills*), for instance one consultant said:

"*For somebody who is normally very well or functional, drives a car, gets out and about and they’ve literally tripped over something and broken a hip, then their rehabilitation is largely going to be the physiotherapist because their needs, otherwise, aren’t so great. For somebody who is much frailer with cognitive impairment and delirium and lives at home and has a lot of functional deficit, then actually the physiotherapist may not have as much a role to play. It may be more occupational therapy and me and the nursing staff*." (P9, female, orthogeriatric consultant, 18 years of experience)

A positive team culture was also enabled by dedicating time to support shared learning within and across professional groups *(Skills*). This learning included both formal (in-service training) and informal (support to extend skills) training which was sometimes evaluated through e.g., audit to enable advocacy for additional resource *(Behavioural regulation),* but often not, as one OT voiced:

“*We’ve given the empowerment, if you like, we don’t have to get a patient up on day zero, nursing staff will do it. So we’ve gone in with them, we’ve taught them, we’ve given them the competencies, they’re competent to do it, they take the same assessments as we do and they can get them up and get them going*.” (P5, female, team lead orthopaedic physiotherapist, 21 years of experience)

Engaging patients and their carers

Promoting a positive attitude towards rehabilitation was considered of particular importance in the context of limited physiotherapist and/or occupational therapy staff resources (*Belief about consequences, Goals*). To reinforce this individual responsibility of the patient, health care professionals often felt they needed to present a unified front to support patients’ independence, by reminding and facilitating this approach to rehabilitation among different team members (*Belief about capabilities, Social/professional role and identity).* For instance, one consultant commented:

“*I explain to patients, part of your rehab isn’t just the time that you spend with the physio or with the OT, it’s also the time walking out to the bathroom with the nurse or the healthcare assistant or even by yourself is a part of your rehab because that’s you starting to use your muscles again and starting to practice your walking etc, that lots of activity that you’re doing in hospital without maybe another person being there with you.”* (P19, female, orthogeriatric consultant, 3.5 years of experience)

All healthcare professionals acknowledged that taking ownership for their early rehabilitation after hip fracture would not be possible for all patients. In particular, the challenge of supporting patients with cognitive impairment to engage in rehabilitation was identified across all professional groups (*Belief about consequences*).

** See reference 30 in main paper
