# Supplementary material for: Multidisciplinary team healthcare professionals’ perceptions of current and optimal acute rehabilitation, a hip fracture example A UK qualitative interview study informed by the Theoretical Domains Framework
Source: PLoS One. 2022 Nov 18;17(11):e0277986. doi: 10.1371/journal.pone.0277986 (PMC9674178; doi:10.1371/journal.pone.0277986)
Supplement: Attachment — Submitted filename: Response to Reviewers.docx [file pone.0277986.s003.docx]

Academic Editor Comments

1. Please ensure that your manuscript meets PLOS ONE's style requirements, including those for file naming.

We have edited and checked style requirements are met.

2. Thank you for stating the following in the Competing Interests section: “UKRI Future Leaders Fellowship funding [Grant Ref: MR/S032819/1] provides salary support for KS and SG. KS is the Chair of the Scientific and Publications Committee of the Falls and Fragility Fracture Audit Programme which managed the National Hip Fracture Database audit at the Royal College of Physicians. ES is supported by NIHR ARC Wessex. GM and KL declare no competing interests.”
   Please confirm that this does not alter your adherence to all PLOS ONE policies on sharing data and materials, by including the following statement: ""This does not alter our adherence to PLOS ONE policies on sharing data and materials.” (as detailed online in our guide for authors http://journals.plos.org/plosone/s/competing-interests). If there are restrictions on sharing of data and/or materials, please state these. Please include your updated Competing Interests statement in your cover letter; we will change the online submission form on your behalf.

An updated Competing Interests statement is now included in the Cover letter.

3. In your Data Availability statement, you have not specified where the minimal data set underlying the results described in your manuscript can be found. PLOS defines a study's minimal data set as the underlying data used to reach the conclusions drawn in the manuscript and any additional data required to replicate the reported study findings in their entirety.

There are restrictions to sharing our data publicly. We have expanded on the details of these restrictions in the Data Availability statement.

Our Data Availability statement now reads:

We did not receive consent from participants to share or archive their personal or anonymised data publicly. Participants agreed to share their anonymised data for dissemination purposes, such as in journal publications and conferences. Therefore, we have made excerpts of the transcripts relevant to our findings available within the paper (in the text of the results and Table 2).

Reviewer #1 Comments

1. Have the authors made all data underlying the findings in their manuscript fully available? Reviewer #1: No

Our Data Availability statement now reads:

We did not receive consent from participants to share or archive their personal or anonymised data publicly. Participants agreed to share their anonymised data for dissemination purposes, such as in journal publications and conferences. Therefore, we have made excerpts of the transcripts relevant to our findings available within the paper (in the text of the results and Table 2).

.

2. First, thank you for the work. The topic is much needed and the Theoretical Domains Framework is an excellent choice to study health care professionals perceptions of current and optimal provision of rehabilitation in the acute hospital setting. It would be useful to include proposed solutions to recruit nurses and physicians (i.e. snowball sampling through included participants). I am curious which efforts you have done to recruit other healthcare professionals and whether they informed you why they did not want to take part in the study (answers given in line with your proposed perception that rehabilitation is a therapist’s role?)

Thank you for agreeing with us on the importance of the topic and choice of framework.

We updated the methods section to read:

We intended to recruit an even proportion of physiotherapists, occupational therapists, nurses, and physicians with at least 2 years experience of working within acute rehabilitation after hip fracture in the UK, or until we reach data saturation (REF). This was to gain insight from a range of different professional groups. By using a convenience sampling approach [27], we recruited 15 hospital sites through relevant societies (Chartered Society of Physiotherapy, Royal College of Occupational Therapists, Royal College of Nursing, and the British Geriatrics Society) and via social media. Potential participants contacted one member of the research team (KL) to express their interest in taking part in the study, receive answers to their questions, and provide informed written consent prior to scheduling their interview. We limited recruitment of physiotherapists and occupational therapists at 14 as no new themes were emerging from these professional groups.

We also updated the limitations section of the discussion to read:

‘We sought to capture a multidisciplinary perspective on rehabilitation however, participation was dominated by physiotherapists and occupational therapists (n =14), those with at least 10-years of experience (n = 14), and who were female (n = 18) despite efforts to recruit other professional groups from multiple sources. This may reflect a perception that rehabilitation is a therapist’s role opposed to a care structure/process[17].’ This may lead to an imbalance of the perspectives of healthcare professionals more broadly limiting generalisability of the findings. Future research may focus on under-represented groups (in terms of profession, experience, and sex) to broaden our understanding of optimal acute rehabilitation from the perspective of more groups. Moreover, alternative sampling strategies, such as snowball sampling, a procedure commonly used to increase the sample diversity of studies among ‘difficult-to-reach’ populations [REF to add– Kirchherr, 2018] may complement future research recruitment strategies.’

3. Additionally, I personally prefer the term person-centered above patient-centered emphasizing to look after a person instead of only the disease/injury which sounds also more in line with the engagement of patients and carers in rehabilitation.

We updated from patient-centered to person-centered throughout the manuscript.

4. Moreover, I wondered if you formulated in- and exclusion criteria for participating in this study. Table 1 can thereby be improved by adding experience of participants with rehabilitation of patients with hip fracturs (e.g. how many seen per year) next to clinical experience. Another small remark on Table 1: Would be nicer to read when it is sorted according to e.g. years of clinical experience.

Table 1 is now sorted by clinical years of experience.

We added a row in Table 1 to indicate the number of hip fractures seen per year next to location.

5. Finally, only two participants were male. Did you have the intention to recruit a certain percentage/distribution of men/women, less vs high experienced professionals? If not, what was the underlying reason? And if yes, why did it not work out?

We updated the methods section to specify:

‘We intended to recruit an even proportion of physiotherapists, occupational therapists, nurses, and physicians with at least 2 years experience of working within acute rehabilitation after hip fracture in the UK’

We did not sample based on sex and have updated the limitations section to read:

‘We sought to capture a multidisciplinary perspective on rehabilitation however, participation was dominated by physiotherapists and occupational therapists (n =14), those with at least 10-years of experience (n = 14), and who were female (n = 18) despite efforts to recruit other professional groups from multiple sources. This may reflect a perception that rehabilitation is a therapist’s role opposed to a care structure/process[17].’ This may lead to an imbalance of the perspectives of healthcare professionals more broadly limiting generalisability of the findings. Future research may focus on under-represented groups (in terms of profession, experience, and sex) to broaden our understanding of optimal acute rehabilitation from the perspective of more groups.’
