# Supplementary material for: Multidisciplinary team healthcare professionals’ perceptions of current and optimal acute rehabilitation, a hip fracture example A UK qualitative interview study informed by the Theoretical Domains Framework
Source: PLoS One. 2022 Nov 18;17(11):e0277986. doi: 10.1371/journal.pone.0277986 (PMC9674178; doi:10.1371/journal.pone.0277986)
Supplement: Attachment — Submitted filename: Response to Reviewers.docx [file pone.0277986.s004.docx]

**Thank you for taking the time to review our manuscript. We have revised the draft based on your comments (inclusive of a review of the consistency in terminology throughout) and believe it to be much improved. All changes are highlighted in yellow on the revised draft.**

1. Abstract: Calling it a qualitative semi-structured interview study – can change please identify the methodology

We updated the abstract methods to read:

*‘A qualitative design was adopted using semi-structured telephone interviews with 20 members of the acute multidisciplinary healthcare team (occupational therapists, physiotherapists, physicians, nurses) working on orthopaedic wards at 15 different hospitals across the UK. Interviews were audio-recorded, transcribed verbatim, anonymised, and then thematically analysed drawing on the Theoretical Domains Framework to enhance our understanding of the findings.’*

2. **Methods: You again call it a qualitative study drawing on the framework – fine but what is the methodology and what is your world view? The framework presumably acted to inform your interviews and structure your analysis this is where the detail is needed.**

We replaced the following paragraph on the TDF from the Study design section to the Introduction:

*‘The domains enable structuring of qualitative data to identify behaviours and implementation barriers and facilitators to target for intervention. Once these determinants of behaviour are identified, they offer a useful framework for the choice of future quality improvement interventions.’*

We also updated sections of the methods to include the following text:

*Study design*

*‘A qualitative design was used to provide an in-depth understanding of multidisciplinary healthcare professionals’ perspectives of current and optimal acute rehabilitation and perceived implementation facilitators and barriers. The study was underpinned by an interpretivist philosophical view of the social world which is based on the premise that our knowledge of reality is socially constructed by our perceptions and interpretations of it.’*

*Data collection*
*‘The topic guide was theoretically informed, with questions and prompts mapped to the TDF to ensure the topic guide would enable generation of data related to individual, social, and environmental determinants of behaviours and implementation barriers as part of this framework (Supplementary File 1).’*

*Data analysis*
*‘Data analysis proceeded until data saturation was deemed to have been reached, in which no new relevant themes were emerging from the qualitative data [27]. A thematic analysis approach was used to analyse and organise themes grounded in the qualitative data [29], drawing on the TDF [25] to enhance our understanding of what behaviours and implementation barriers and facilitators were perceived to influence optimal rehabilitation in acute hospital settings.*

*Specifically, the qualitative analysis process involved a number of phases. The first phase involved three authors (SG, GM, KL) reading all transcripts, generating initial themes (codes), and grouping similar themes together (initial and axial coding) in NVivo (version 12) [29]. In the second phase these clusters of codes were used to organise initial themes into conceptual themes and related subthemes using the ‘one sheet of paper method’ approach, whereby similar and diverse perspectives among participants were identified across different professional groups [30]. The final phase involved mapping the findings within each theme to the TDF domains to identify behaviours and implementation barriers and facilitators perceived to influence optimal rehabilitation in acute hospital settings (see Supplementary file 2 for an example). These themes were refined iteratively with discussions within the research group.’*

3. **Can you confirm the eligibility criteria was just at least 2 years experience? Was there no exclusion criteria? Please separate a section on sampling and sample size from eligibility criteria. Also may be consider putting line 119 start around contacting participants into data collection section**

We updated the Eligibility and Sampling sections to read:

*Eligibility criteria*

*‘We aimed to recruit multidisciplinary team healthcare professionals, including physiotherapists, occupational therapists, nurses, and physicians with at least 2 years experience of working within acute rehabilitation after hip fracture in the UK [27]. There was no additional inclusion or exclusion criteria.’ This was in order to gain insight from a range of different professional groups.’*

*Sampling and recruitment*

*‘We used a convenience sampling approach [28] to recruit multidisciplinary team healthcare professionals by advertising the study through relevant professional societies (Chartered Society of Physiotherapy, Royal College of Occupational Therapists, Royal College of Nursing, and the British Geriatrics Society) and via Twitter.’*

*Data collection*

*‘Potential participants contacted one member of the research team (KL) by email to express their interest in taking part in the study, receive the participant information sheet and consent form, and ask questions. Interested participants return signed consent forms by email. ‘Individual semi-structured telephone interviews were conducted by one author (KL). [...]’*

4. **Remove [28] from line 124 – not sure why it is there**

Apologies, this was a mistake which we have now corrected.

5. **Please add an audit trail for the reader to understand what you did – for instance we need a reference for your inductive thematic analysis which suggests an initial process of open coding? Correct? Why not use a framework analysis. Can you be clear on what you mean by aid interpretation of findings – you mean you used it as a framework for major themes then identified minor themes? You say initial themes – to be clear you mean themes from open coding or from the framework? Please talk about phases and reference a supplementary file for examples of each phase. When you mention reference 31 again it is hard to follow what you have done.**

We updated the data analysis section. Please see the updated paragraph in the response to comment 2.

We also added a supplementary file (S2 Appendix I) with examples of each phase of our analysis.

6. **You mention saturation again here – given how big the TDF is I am surprised that you have captured all domains but fine.**

We updated the data analysis section to read:

*‘Data analysis proceeded until data saturation was deemed to have been reached, in which no new relevant themes were emerging from the qualitative data [27]. A thematic analysis approach was used to analyse and organise themes grounded in the qualitative data [29], drawing on the TDF [25] to enhance our understanding of what behaviours and implementation barriers and facilitators were perceived to influence optimal rehabilitation in acute hospital settings.’*

7. **You identify information regarding reflexivity but need to talk about how you enhanced quality and what you consider as quality based on your world view.**

We updated the text to read:

*‘Thus, we considered the interdisciplinary nature of the research team enhanced quality in this study because the team brought together multiple perspectives to understand how acute rehabilitation after hip fracture could be optimised based on multidisciplinary team healthcare professionals’ perceptions. This aligned with our interpretivist philosophical view of reality as socially constructed.’*
